# A comparison of sotagliflozin therapy for diabetes mellitus between week 24 with week 52: A protocol for mixed treatment comparisons meta-analysis

**DOI:** 10.1097/MD.0000000000017976

**Published:** 2019-11-22

**Authors:** Nie Zhang, Zhi-Qun Gu, Yun-Long Ding, Liu Yang, Mao-Bing Chen, Qi-Han Zheng

**Affiliations:** a Department of Endocrinology; b Department of Neurology, Jingjiang People's Hospital, The Seventh Affiliated Hospital of Yangzhou University; c Department of Emergency, Wujin Hospital Affiliated with Jiangsu University, and the Wujin Clinical College of Xuzhou Medical University, Changzhou Jiangsu, PR China.

**Keywords:** diabetes mellitus, meta-analysis, randomized controlled trial, SGLT-1 inhibitors, SGLT-2 inhibitors, sotagliflozin

## Abstract

**Background:**

According to the centers for disease control and prevention, 14% of American adults have diabetes – 10% know it, and more than 4% go undiagnosed. Sotagliflozin is a new type of diabetes drug This study is to compare the efficacy of Sotagliflozin therapy for Diabetes Mellitus (DM) between week 24 with week 52.

**Methods and analysis:**

Through to October 2019, Web of Science, PubMed Database, Cochrane Library, EMBASE, Clinical Trials and CNKI will be searched to identify randomized controlled trials (RCTs) exploring SOTA therapy for DM. Strict screening and quality evaluation will be performed on the obtained literature independently by 2 researchers; outcome indexes will be extracted. The bias risk of the included studies will be evaluated based on Cochrane assessment tool. Meta-analysis will be performed on the data using Revman 5.3 software. We will provide practical and targeted results assessing the lost efficacy of SOTA therapy for DM from week 24 to week 52, to provide reference for clinicians.

**Ethics and dissemination:**

The stronger evidence about the lost efficacy of SOTA for DM from week 24 to week 52 will be provided for clinicians.

**Trial registration number:**

PROSPERO CRD42019133027.

**Strengths and limitations of this study:**

Whether the efficacy of SOTA could last for a long time is still inconclusive, high quality research is still lacking, and this study attempts to explore this issue; The efficacy of SOTA at different times will be compared by direct comparisons and indirect comparisons, this can lead to more accurate and reliable results; The quality of the included literatures are uneven, and some data might be estimated by calculation, which may affect the quality of this study.

## 1 Introduction

Diabetes mellitus (DM), commonly known as diabetes, is a group of metabolic disorders characterized by high blood sugar levels over a prolonged period.^[1]^ Main symptoms include frequent urination, increased thirst, increased hunger, and emaciation.^[2]^ If be treated not in time, diabetes can cause many complications,^[3,4]^ including diabetic ketoacidosis (DKA), hyperosmolar hyperglycemic state (HHS), or death.^[5]^ Serious long-term complications include cardiovascular disease, stroke, chronic kidney disease, foot ulcers, and damage to the eyes.^[6,7]^

As of 2017, an estimated 425 million people had diabetes worldwide,^[8,9]^ with type 2 diabetes making up about 90% of the cases.^[4]^ This represents 8.8% of the adult population both men and women.^[10]^ Trend suggests that rates will continue to rise.^[11]^ Diabetes at least doubles a person's risk of early death. In 2017, diabetes resulted in approximately 3.2 to 5.0 million deaths.^[9]^

Sotagliflozin (SOTA) is a novel SGLT-1/SGLT-2 dual inhibitor.^[12–14]^ Relying on its unique hypoglycemic mechanism, it reduces the absorption of glucose in the gastrointestinal tract by inhibiting SGLT-1 and increases the excretion of glucose by the kidneys by inhibiting SGLT-2.^[15–17]^ SOTA was approved in EU for Adults With Type 1 Diabetes, so it can be used to treat Type 1 Diabetes Mellitus (T1DM) and Type 2 Diabetes Mellitus (T2DM).^[18]^ But it seems that its ability to reduce blood sugar levels decreases over time. This study try to find out the long-term therapeutic effect of SOTA.^[19–22]^

## 2 Methods

### 2.1 Design and registration

A meta-analysis will be conducted to evaluate the comparison of Sotagliflozin therapy for Diabetes Mellitus between week 24 with week 52. This protocol has been registered on the international prospective register of systematic reviews (PROSPERO), registration number: CRD42019133027 (https://www.crd.york.ac.uk/PROSPERO). No ethical approval is required since this study used data that were already in the public domain.

### 2.2 Study selection

#### 2.2.1 Study type

This study will include Randomized Controlled Trials (RCTs).

#### 2.2.2 Study object

Limitations of study object included in the analysis:

(1) patients with a definite diagnosis of DM;
(2) without serious diabetes complications;
(3) without cancer, heart failure, respiratory failure and other underlying diseases.

#### 2.2.3 Intervening measure

Patients received treatment for a period of time to stabilize their blood glucose and glycosylated hemoglobin (HbAlc) prior to the experiment. SOTA (200 mg or 400 mg) or placebo should be taken once a day.

#### 2.2.4 Outcome indicator

The following outcomes will be assessed compared:

(1) differences in HbAlc,
(2) differences in weight,
(3) differences in fasting blood glucose,
(4) differences in 2-hour postprandial blood glucose,
(5) differences in the rate of well-controlled diabetes (HbAlc < 7 after the end of the study, and no serious complications).

#### 2.2.5 Exclusion criteria

Literature whose data cannot be extracted or utilized; literature on animal experiments; literature reviews, etc.

### 2.3 Data sources and searches

**Figure 1:**
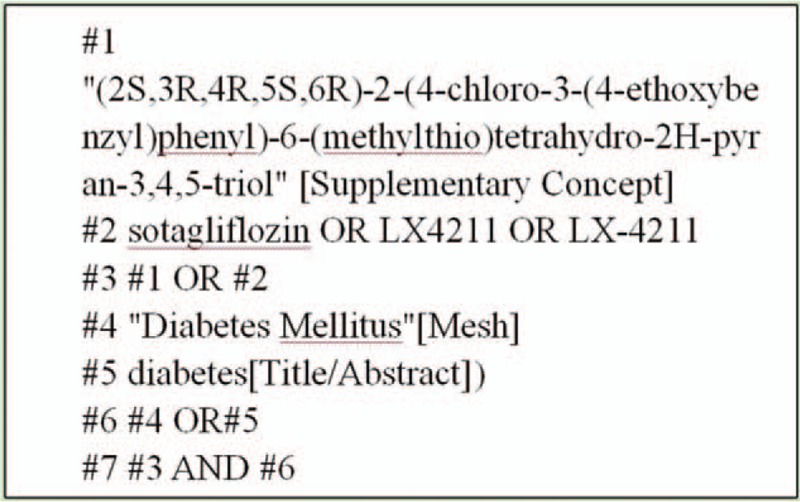
PubMed database retrieval strategy.

We will search English and Chinese language publications through October 2019 using the following databases: Web of Science, PubMed, Cochrane Library, Embase, Clinical Trails and the China National Knowledge Infrastructure (CNKI). Search terms were “sotagliflozin”, “Diabetes Mellitus”,“LX4211” and so on. Here, we use the PubMed database as an example (see Fig. 1).

### 2.4 Study screening, data extraction and risk assessment of bias

Data will be collected independently by two researchers. The unqualified studies will be eliminated, and the qualified ones will be screened out after reading the title, abstract and full text. Then, the research data were extracted and checked, and disagreements were discussed or a decision was made by the author. The extracted data included the following:

1. basic information of the study, including title, author, and year of publication;
2. characteristics of the included study, consisting of study duration, sample size of test group and control group, and intervention measures;
3. outcome indicators and data included; and
4. collection of risk assessment elements of bias.

The risk of bias in the included studies will be assessed by using the RCT bias risk assessment tool recommended in the Cochrane Handbook for Systematic Reviews of Interventions (5.1.0).

### 2.5 Statistical analysis

RevMan 5.3 software will be used for the meta-analysis. The dichotomous variables will be relative risk (RR) as effect indicators, the continuous variables are expressed as mean difference (MD) as effect indicators, and the estimated value and 95% confidence interval (CI) will be included as effect analysis statistics. A heterogeneity test will be conducted with the results of each study. The fixed effect model will be used for analysis if there was no statistical heterogeneity between the results (I^2^ ≤ 50%). The sources of heterogeneity need to be analyzed if there was statistical heterogeneity between the results (I^2^ > 50%). After excluding the influence of obvious clinical heterogeneity, the random effect model will used for analysis. The significance level is set α = 0.05.

### 2.6 Mixed treatment comparisons meta-analysis

**Figure 2:**
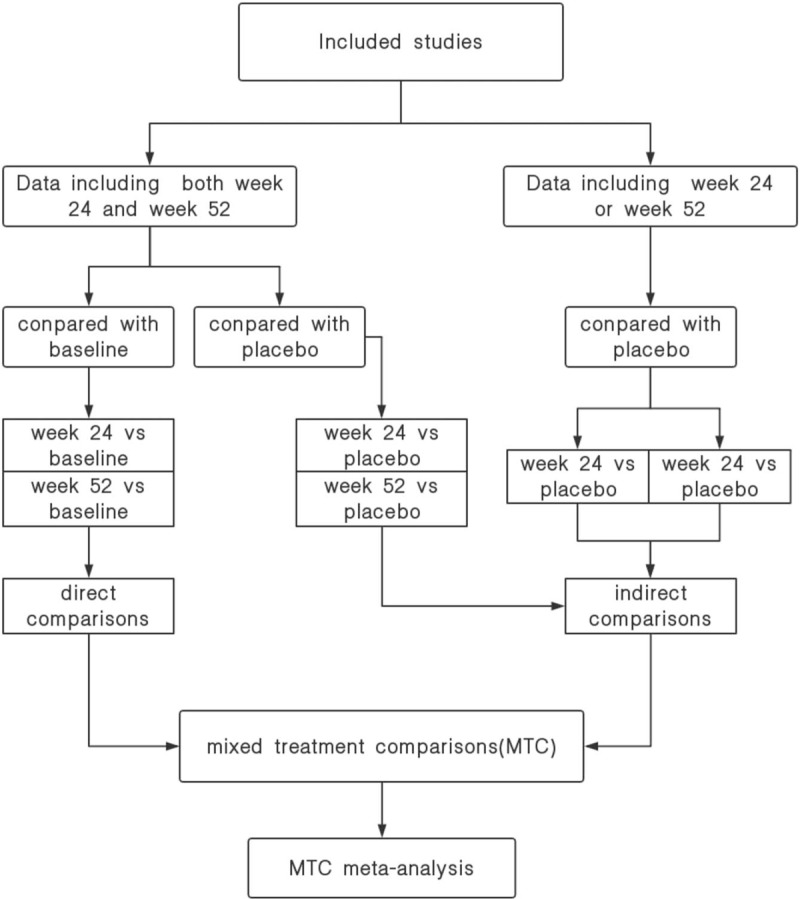
Researches classification process.

The included studies will be classified into direct comparisons and indirect comparisons. Indirect comparisons between week 24 with week 52 will be calculated through common placebo. Direct comparisons and indirect comparisons data are obtained through meta-analysis, and inverse variance method is adopted to merge (see Fig. 2).

### 2.7 Subgroup analysis

Subgroups will be established based on difference of oral dose (400 mg/day, 200 mg/day) and the type of DM (T1DM, T2DM).

### 2.8 Assessment of publication bias

If more than 10 articles are available for quantitative analysis, we will generate funnel plots to assess publication bias. A symmetrical distribution of funnel plot data indicates that there is no publication bias, otherwise, we will analyze the possible cause and give reasonable interpretation for asymmetric funnel plots.

### 2.9 Confidence in cumulative evidence

GRADE system will be used for assessing the quality of our evidence. According to the grading system, the level of evidence will be rated high, moderate, low, and very low.^[23]^

## 3 Discussion

SOTA is a new generation SGLT inhibitor that can act on both SGLT-1 and SGLT-2. SGLT-1 is mainly expressed in the small intestine and kidneys and is responsible for transporting glucose and galactose in the small intestine and reabsorbing glucose in the proximal convoluted tubules. SGLT-2 is specifically located in the proximal convoluted tubules of the kidney and is responsible for the renal reabsorption of glucose in the urine and is responsible for approximately 90% of glucose reabsorption.^[24]^

Prior to starting this study, reading literatures found that its therapeutic effect curve is always high in early stage and low in late stage. These differences may have statistically significant. But right now there are few studies paying close attention to it. DM is a disease that requires long-term treatment. If SOTA's effectiveness continues to decline, the dosage should be adjusted or other drugs should be added to the treatment.

There are two methods to gather data. One we only collect studies that treatment duration longer than 52 weeks, and studies must including the data at week 24 and week 52. In this way, the data are comparable, and have fewer confounding factors. But it will lost a lot of studies. Or we might collect any studies including the data at week 24 or week 52. Because sensitivity to drugs varies among different populations, if there are not enough studies, the differences might make these data uncomparable. The more studies or multicenter researches there are, the more comparable the data are. In this study, we will choose the second method. Indirect comparisons combined with direct comparisons will make the results more reliable.

This study will conduct a mixed treatment comparisons meta-analysis of related RCTs, and provide evidence on the comparison of SOTA therapy for Diabetes Mellitus between week 24 with week 52, so as to better guide clinical practice.

## Author contributions

Nie Zhang, Zhiqun Gu, Liu Yang and Yunlong Ding designed the systematic review. The protocol was drafted by Nie Zhang and revised by Zhiqun Gu and Liu Yang. Maobing Chen and Qihan Zheng developed the search strategy. Nie Zhang, Zhiqun Gu, Liu Yang, Yunlong Ding, Maobing Chen and Qihan Zheng will independently work on study selection, quality assessment, data extraction, and synthesis.
